# Enhanced Grätzel Solar Cells Using Carbon Nanodots and Natural Dye

**DOI:** 10.1021/acsphyschemau.4c00080

**Published:** 2024-12-16

**Authors:** Moisés do Amaral Amancio, Yonny Romaguera Barcelay, Ariamna Gandarilla, Ronald Rastre Sales, Thiago Monteiro de Souza, Francisco Xavier Nobre, Ellen Raphael, Walter Ricardo Brito

**Affiliations:** † LABEL—Laboratório de Bioeletrônica e Eletroanalítica (LABEL), Department of Chemistry, Federal University of Amazonas, Manaus 69067-005, Amazonas, Brazil; ‡ BioMark@UC/CEMMPRE-ARISE, Department of Chemical Engineering, Faculty of Science and Technology, University of Coimbra, Coimbra 3030-790, Portugal; § Department of Chemistry, Environment, and Food (DQA), Group of Energy Resources and Nanomaterials (GREEN Group), Federal Institute of Education, Science and Technology of Amazonas, Campus Manaus Centro, Manaus 69020-120, Amazonas, Brazil; ∥ FENTOMLAB, School of Technology, University of the State of Amazonas, Av. Darcy Vagas, 1200, Parque Dez de Novembro, Manaus 69050-020, Amazonas, Brazil

**Keywords:** CNDs, natural dye, electrolyte, triiodide (I_3_^−^) diffusion, DSSCs

## Abstract

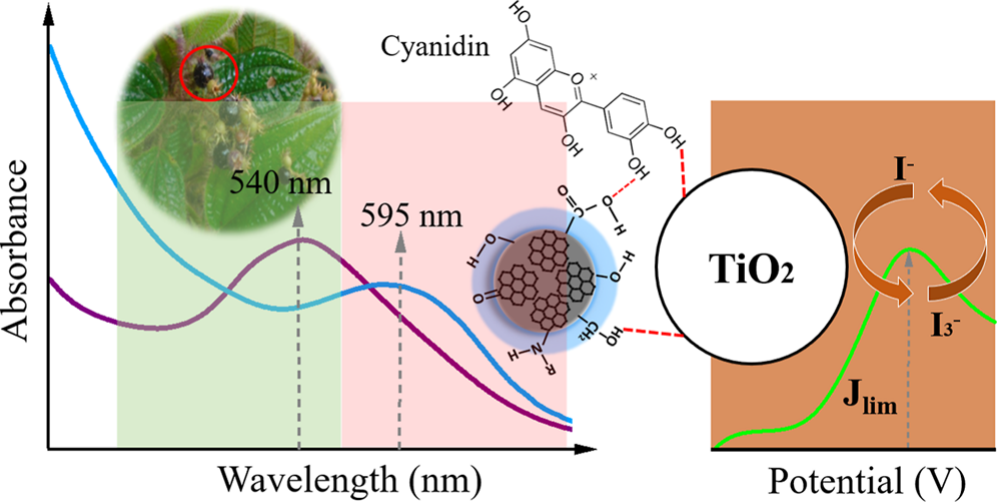

Photoluminescent carbon nanodots have shown great potential in various scientific fields, with prominence in technological applications. Their low toxicity, affordability, and biocompatibility make them a promising alternative in developing next-generation solar cells. This study explored carbon nanodots (CNDs) as an alternative to traditional carbon allotropes, focusing on creating sustainable and environmentally friendly Grätzel-type solar cells using low-cost materials. The feasibility of CNDs, in conjunction with *Leandra australis* fruit dye as TiO_2_ sensitizers, was investigated, as well as the impact on the diffusion coefficient of I_3_^–^ in the electrolyte due to excess I_2_. The synergistic interaction between the dye and CNDs altered the material energy states, red-shifting the solution’s light absorption region (Dye-CNDs). Improved *V*_oc_ and *J*_sc_ values were recorded, and as a result, a 5% increase in energy conversion efficiency (η) was calculated for the FTO/TiO_2_-Dye-CNDs photoanode cell compared to the control photoanode cell (FTO/TiO_2_-Dye). These results highlight the promising potential of CNDs as a low-cost alternative to significantly enhance the potential of Grätzel-type solar cells, paving the way for more sustainable energy solutions.

## 1 Introduction

The pursuit of photovoltaic devices that achieve high energy conversion efficiency while maintaining low production costs remains a key focus within the scientific community. Among such technologies, Grätzel-type solar cells, commonly called dye-sensitized solar cells (DSSCs), have emerged as a promising and cost-effective option for sustainable energy generation.^1,2^ These cells employ a photoactive layer that can be sensitized using synthetic and naturally derived dyes, including extracts from various plants and fruits, thereby enhancing environmental compatibility.^3,4^ Although synthetic dyes offer favorable properties and reliable energy conversion efficiencies, their reliance on rare or heavy metals and inherent toxicity concerns pose significant environmental and health risks, limiting broader applicability.^5,6^

Recent developments in nanoscience and nanotechnology have led to the discovery of new materials with significant potential for DSSC applications. Carbon-based nanomaterials, including graphene quantum dots (GQDs), carbon nanotubes (CNTs), and photoluminescent carbon nanodots (CNDs), also known as carbon quantum dots, exhibit remarkable optical, electronic, and structural properties, positioning them as strong candidates for use in sustainable DSSC designs.^7−9^

Among the various carbon allotropes, carbon nanodots (CNDs) are readily obtainable, cost-effective, nontoxic, and biocompatible graphitic nanomaterials suitable for diverse environmental applications.^8,10,11^ Due to their inherent low toxicity and distinctive optical properties, CNDs have found utility across various technological applications, including sensors, bioimaging, drug delivery, photodynamic therapy, photocatalysis, light-emitting devices, and photovoltaic systems.^12−16^ Morphologically, CNDs are photoluminescent graphitic nanoparticles, typically smaller than 10 nm, primarily composed of sp^2^ hybridized carbon functionalized with polar groups like carboxylic acids (−COOH) and hydroxyls (−OH). This functionalization imparts CNDs with a strong capacity to form hydrogen bonds with various molecules, a property crucial for their effective incorporation into dye-sensitized solar cells (DSSCs).^17,18^

DSSCs have undergone extensive optimization efforts targeting improvements to the photoanode, electrolyte, and counter electrode, establishing themselves as one of the most promising cost-effective photovoltaic technologies in recent years.^19^ Recent advancements underscore that long-term stability and suboptimal conversion efficiency issues can be mitigated using highly effective photoactive materials and electrolytes. These components enhance the regeneration of photoactive dyes and facilitate electron recombination at the counter electrode.^20^ However, concerns surrounding synthetic dyes, often derived from heavy metals or complex organic compounds, persist due to their potential for environmental toxicity, bioaccumulation, and negative ecological impact.^5^ To address these challenges, natural dyes from plants and fruits present a compelling and environmentally friendly alternative for developing efficient and sustainable photovoltaic devices. Integrating these natural dyes with materials capable of modulating their photochemical properties represents a promising strategy for creating low-cost, high-efficiency DSSCs.

This study uses the hydrothermal method to detail the green synthesis of carbon nanodots derived from the Pixirica fruit (*Leandra australis*). The feasibility of employing these CNDs in combination with a natural dye as a photosensitizer for Grätzel-type solar cells was explored. Additionally, we conducted studies to optimize the electrolyte composition (I^–^/I_3_^–^) by determining the ideal concentration of I_2_, thereby improving the diffusion coefficient, and enhancing the stability and performance of the cells. Our results demonstrate that integrating CNDs with natural dye from *L. australis* leads to modifying the energy states within the material (Dye-CNDs), broadening the light absorption range. This integration also reduced the photoanode structure’s charge transfer resistance (*R*_ct2_) (FTO/TiO_2_-Dye-CNDs). Consequently, enhanced short-circuit current densities (*J*_sc_) and a 5% increase in overall energy conversion efficiency were observed compared to photoanodes utilizing only the natural dye (FTO/TiO_2_-Dye).

## 2 Materials and Methods

### 2.1 Materials

In this work, the following materials and reagents were used: acetone (99.5%), isopropanol (99.8%), acetonitrile (99.0%), ethylene glycol (99%), iodine (I_2_), and potassium iodide (99.5%), purchased from Synth. Solaronix TiO_2_ paste and FTO (fluorine-doped tin oxide coated glass) substrates with a surface resistivity of 15 Ω/sq were obtained from MSE Supplies LLC. Scotch, double-sided transparent tape, and 2B pencil from (TRIS Basic). All solutions were prepared with Milli-Q water.

### 2.2 Carbon Nanodots Synthesis

Photoluminescent CNDs were obtained by hydrothermal synthesis, and purple dye from the Pixirica fruit (*L. australis*) was used as a carbon source. 10 g of fruit were extracted with 100 mL of Milli-Q water. Later, the solution was transferred to the hydrothermal reactor and heated in a muffle furnace at 180 °C for 5 h. The dispersion of CNDs was obtained after the removal of carbonaceous slag via centrifugation (20 × 10^3^ rpm/min) for 5 min. The final solution was used to photosensitize the TiO_2_ layer of the cell.

### 2.3 Diffusion of Triiodide (I_3_^–^) Studies

The electrolyte was characterized through linear sweep voltammetry, conducted at a scan rate of 10 mV/s in a symmetrical cell configuration comprising two fluorine-doped tin oxide (FTO) electrodes separated by a 0.1 mm thick O-ring. Detailed insights into the diffusion processes of the electrolyte solution, specifically the I^–^/I_3_^–^ redox couple, can be referenced from the work of Hauch and Georg (2001).^21^ Their study highlights that, due to a substantial excess of iodide ions (I^–^) present in the electrolyte, the diffusion of triiodide ions (I_3_^–^) becomes the primary factor limiting the current density within the cell.^21^ The present study evaluated the diffusion of triiodide ions I_3_^–^(*D*_I_3_^–^_) by determining the diffusion-limited current density (*J*_lim_), calculated following eq 1.
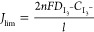
1where *F* is Faraday’s constant (96485.3321 C/mol), *C* is the initial concentration of iodine, *l* is the distance between the two electrodes (here = 0.1 mm), and *n* is the number of electron transfers (here = 2).^22^ Electrolytes were prepared with potassium iodide (KI) and metallic iodine (I_2_) in acetonitrile and ethylene glycol (3:1 v/v). The concentration of I_2_ was varied to explore the ideal concentration for use in the dye cell. The details are shown in Table 1.

**Table 1:** Molar Concentration of I_2_ in the Electrolyte

solvents	KI concentration (mol/L)	I_2_ concentration (mol/L)	electrolytes/symbols
acetonitrile/ethylene glycol	0.6	0.02	E_1_
		0.04	E_2_
		0.06	E_3_
		0.08	E_4_
		0.1	E_5_

### 2.4 Photovoltaic Device

The photovoltaic device was assembled by overlapping the photoanode onto the counter electrode, spaced by a transparent 3 M double-sided Scotch tape intermediated by the electrolyte, the redox pair I^–^/I_3_^–^. The active area of the cell was delimited at 0.25 cm^2^. First, the FTO substrates were cleaned in an ultrasonic bath with acetone, isopropyl alcohol, neutral detergent, solution, and Milli-Q water successively. The photoanode was prepared by depositing TiO_2_ paste (*Solaronix*) on the FTO substrate using the doctor blade technique and following the treatment at 400 °C for 20 min. After this step, the TiO_2_ films were photosensitized via immersion in the respective solutions: natural dye (TiO_2_-Dye) and CNDs dispersion (TiO_2_-Dye-CNDs). The counter electrode (CE) was formed by an FTO substrate (1 × 1.5 cm), and a graphite film using a 2B pencil (TRIS Basic) to enhance charge mobility on the CE surface.

### 2.5 Characterizations

Ultraviolet–visible (UV–vis) absorption spectra were recorded using a Thermo Scientific Evolution 201/220 spectrophotometer. Transmission electron microscopy (TEM) images were acquired with a JEOL JEM-1400 Flash microscope. High-resolution scanning electron microscopy (SEM) images were obtained using a JEOL JSM IT500-HR microscope. ImageJ software (public domain) was used to measure the average size of these nanoparticles.

X-ray diffraction (XRD) patterns were collected with a Bruker D2 Phaser diffractometer, utilizing copper Kα radiation (Cu Kα = 1.5406 Å) at a current of 15 mA. The diffraction data were collected over a 2θ range of 10° to 80°, with a step size of 0.02° and a scan speed of 2° min^–1^.

Photoluminescence (PL) spectra were measured using a SHIMADZU spectrofluorometer, Model RF-5301 PC. Dynamic light scattering (DLS) analysis was performed to estimate the size of dispersed particles using a Litesizer 500 particle analyzer for dispersions and solutions. Fourier transform infrared (FTIR) spectra were acquired using an IV IRTracer-100 spectrometer with an attenuated total reflectance (ATR) module. Photoelectrochemical measurements of the cells were conducted with a PGSTAT128N potentiostat (Metrohm Autolab) and an ORIEL LSC 100 solar simulator under standard light conditions of 100 mW/cm^2^ (AM 1.5G). Equations 2–4 were applied to calculate the fill factor (FF) of the photovoltaic cell, the energy conversion efficiency (PCE) and the electron diffusion length (*L*_n_) value, respectively.^23,24^
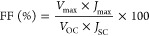
2
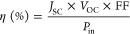
3
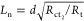
4

## 3 Results and Discussion

### 3.1 CND Structural Characterization

**Figure 1:**
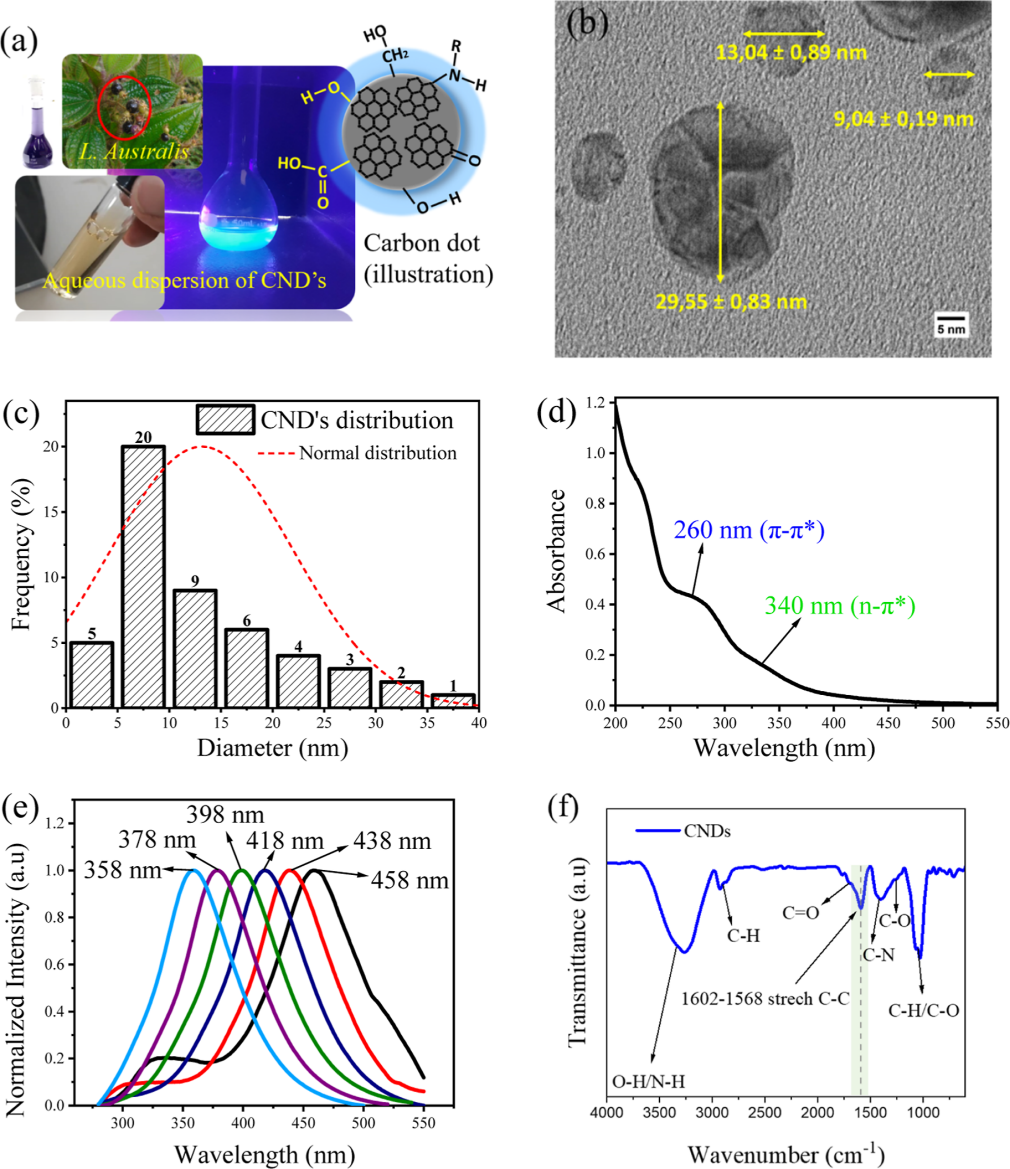
(a) Illustration of CNDs obtained from *L. australis* fruit. (b) Image of CNDs recorded by TEM; (c) size distribution by DLS; (d) UV–vis spectrum; (e) PL spectra at different excitation wavelengths; and (f) ATR-FTIR collected between 650 and 4000 cm^–1^.

The Pixirica fruit dye was a carbon precursor to synthesize photoluminescent carbon nanodots (CNDs). The aqueous dispersion of CNDs, depicted in Figure 1a, demonstrated high stability, and emitted a strong blue fluorescence under ultraviolet light exposure. A structural model of a typical CNDs is also shown in Figure 1a, following established descriptions in recent literature.^25,26^ The transmission electron microscopy (TEM) image of the synthesized CNDs, presented in Figure 1b, reveals monodispersed nanoparticles with a predominantly spherical morphology and a range of diameters. Measured particle sizes varied from 9.04 ± 0.19 to 29.55 ± 0.83 nm. Although characteristic lattice planes for carbon layers, commonly spaced between 0.18 and 0.24 nm, were not distinctly visible,^27^ the overall morphology aligns with the expected characteristics for CNDs. Dynamic light scattering (DLS) analysis, as shown in Figure 1c, revealed a higher density of particles with average sizes predominantly between 5 and 10 nm. The absorption spectrum of the CNDs, shown in Figure 1d, exhibited notable absorption peaks at 260 and 340 nm. The absorption at 260 nm is attributed to π–π* transitions within the graphitic core (C=C bonds). In contrast, the peak at 340 nm corresponds to n–π* transitions, associated with functional groups containing lone electron pairs.^28,29^

The photoluminescence (PL) analysis presented in Figure 1e demonstrates that the emission of CNDs varies with the excitation wavelength. CNDs typically exhibit a stable PL band within the blue spectral region.^30^ This wavelength-dependent emission behavior reflects the intrinsic nature of CND dispersions, which consist of a heterogeneous mixture of graphitic nanoparticles exhibiting varying oxidation states.^31^

The functional groups in the graphitic nanoparticles were analyzed using ATR-FTIR, as shown in Figure 1f. The bands observed at 3200–3600, 1711, and 1250 cm^–1^ correspond to the stretching vibrations of O–H/N–H, C=O, and C–O bonds, respectively, indicating the presence of carboxylic acid and hydroxyl groups in the nanoparticles.^32^ Additionally, the bands near 1599 and 1410 cm^–1^ are attributed to C–C stretching, vibration, and C–N.^33^ The stretching vibrations in this region between 1602 and 1568 cm^–1^ (C–C), are characteristic of these carbon allotropes, as shown in the work of Glória (2021) for studies with multiwalled carbon nanotubes (MWCNTs), highlighted in Figure 1f and used as a certificate of the presence of this graphitic nanomaterial (CNDs).^34^ The bands observed around 2900 and 1040 cm^–1^ correspond to C–H bonds and symmetric and asymmetric CH_3_/bending vibrations, indicating alkyl groups.^35^ The diverse array of functional groups suggests a strong potential for intermolecular interactions, such as hydrogen bonding, with TiO_2_ and the dye, which may significantly impact the properties and performance of the solar cells.^36,37^

**Figure 2:**
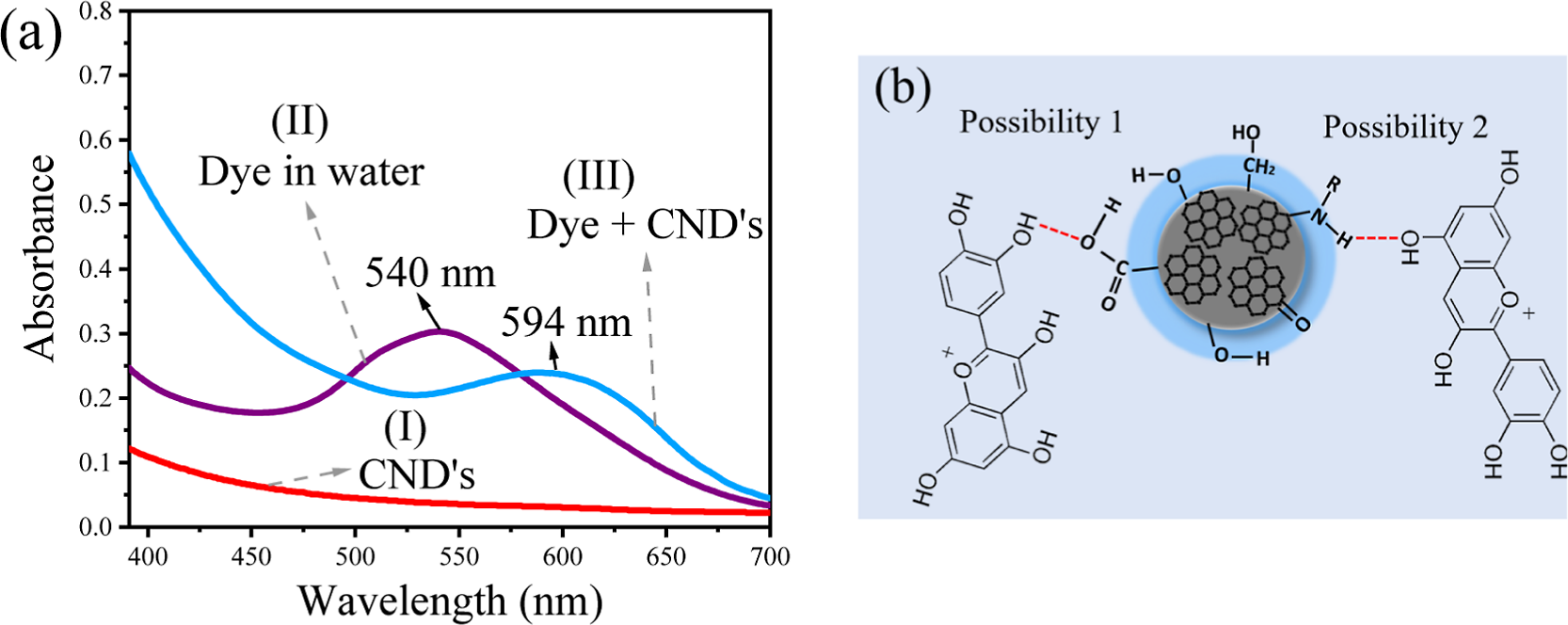
(a) UV–vis spectra: CNDs dispersion (I), dye in water (II), and dye extracted in CNDs dispersion solution (III); (b) illustrative representation of hydrogen bonding interactions between an organic compound from the anthocyanin group and CNDs.

Figure 2a presents the visible absorption spectra for the CNDs dispersion (I), *L. australis* fruit pigment solution in water (II), and the pigment combined within the CNDs dispersion (III). The maximum absorption for the fruit pigment in water (II) was observed at 556 nm, consistent with the characteristic absorption of compounds within the anthocyanin group, commonly found in natural pigments.^38^ In contrast, CNDs exhibit a broad absorption range spanning the entire visible spectrum due to multiple chromophores, as shown in the spectrum (I) of Figure 2a. However, these transitions are associated with forbidden n–π* electronic transitions, resulting in a typically broad and low-intensity absorption profile with no distinctly defined regions.^39^

Consequently, the isolated contribution of CNDs to charge photoinjection within the cell is minimal, necessitating enhancements to increase visible region absorption through modifications or alterations of this component’s properties. Nonetheless, CNDs are critical in various vital processes within the cell. They facilitate efficient charge separation and promote electron transfer to the TiO_2_ conduction band.^40^ Moreover, CNDs contribute to increasing the optical path length via light scattering effects^41^ and enhance the lifetime of excitons generated in the dye, thereby increasing the likelihood of electron transfer to the TiO_2_ substrate.^42^

In Figure 2a(III), substituting water with a CNDs dispersion during the natural pigment extraction process led to notable modifications in the absorption properties of the isolated pigment. The resulting Dye-CNDs combination (III) exhibited a bathochromic shift in its absorption band. This shift can be attributed to structural modifications of the chromophores, resulting from interactions between unsaturated molecules containing oxygen and hydrogen atoms within the composite material.^43^ Alternatively, altering energy states within the composite structure may explain this shift.^44,45^

As detailed by Garlet (2019), the fruit of *L. australis* is characterized by a high anthocyanin content.^46^Figure 2b illustrates potential hydrogen bonding interactions between cyanidin, an organic compound belonging to the anthocyanin group, and a theoretical model representing CNDs. Due to functional groups such as hydroxyls, carbonyls, and amines, CNDs can form hydrogen bonds with other compounds. Two potential bonding scenarios are presented in Figure 2b. In the first scenario, an acidic hydrogen atom of cyanidin forms a hydrogen bond with the oxygen atom of the carboxyl group on the CNDs. In the second scenario, the amine group’s acidic hydrogen bonds with the hydroxyl group’s oxygen atom in cyanidin. This diverse array of functional groups presents in both cyanidin, and CNDs facilitates the formation of a complex hydrogen bonding network, thereby significantly influencing the composite material’s optical, electronic, and structural properties.

The potential to broaden the absorption band of charge photoinjector materials is highly advantageous for dye-sensitized solar cells (DSSCs) applications. Expanding this band enables the material to capture and utilize a broader region of the incident light spectrum, thereby enhancing the generation of electron–hole pairs and increasing the charge density within the conduction band (CB) of the semiconductor. Such improvements directly translate to greater energy conversion efficiency, as the solar cells can generate more electrical power from the same light exposure.^47^ Additionally, a higher rate of photogenerated charge carriers and a reduced recombination rate further optimize cell performance, resulting in superior efficiencies in light-to-energy conversion.^48^

### 3.2 Diffusion of Triiodide (I_3_^–^)

**Figure 3:**
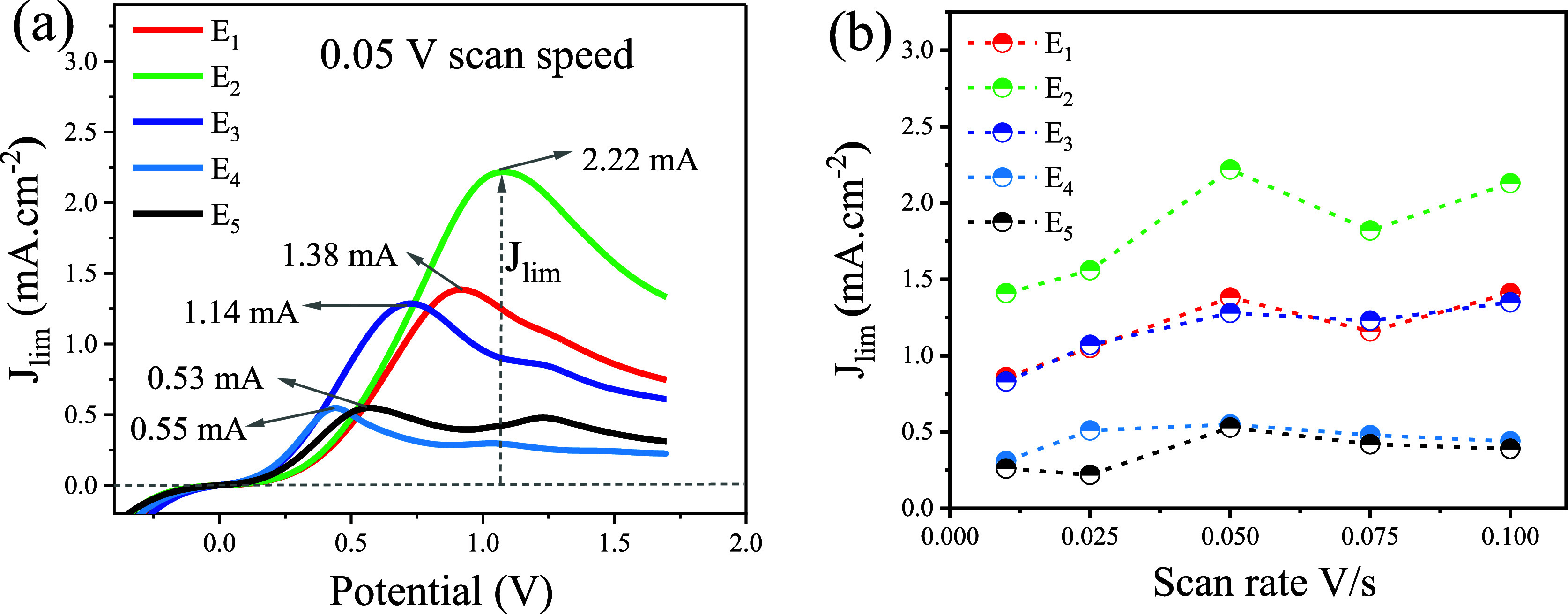
Symmetrical cell–Linear sweep voltammetry: (a) diffusion–imited current density (*J*_lim_) at 0.05 V/s; (b) *J*_lim_ varying the scan rate from 0.01 to 1.0 V/s.

Figure 3a displays the linear sweep voltammetry profiles emphasizing the maximum diffusion-limited current density (*J*_lim_) values at a sweep speed of 0.05 v/s. Figure 3b shows the maximum current density values varying the scanning speed from 0.01 to 1.0 V/s. Among the electrolytes investigated, composition E_2_ presented the highest *J*_lim_ value, reaching 2.22 mA cm^–2^ at a scanning speed of 0.05 V/s. It is observed that with the increase in the concentration of I_2_ in the composition of the electrolytes, the *J*_lim_ values decrease regardless of the scanning speed, showing that the diffusional process of I_3_^–^ strongly depends on the concentration of I_2_ in the solution.^21^

These electrolyte diffusion constants (*D*_I_3_^–^_) were determined using eq 1. Given the surplus of I^–^ ions in the electrolyte, the diffusion of I_3_^–^ emerges as the primary factor limiting the current in the cell. Consequently, the recorded diffusion-limited current density *J*_lim_ is directly proportional to the diffusion process of I_3_^–^ in the electrolyte (*D*_I_3_^–^_).^21^

The *D*_I_3_^–^_ values are shown in Table 2. At the I_2_ concentrations evaluated, the electrolytes remained within the limiting value of the diffusion constant for these electrolytes, which is at least 10^–6^ cm^2^ s^–1^.^21,49^ However, this study calculated the highest diffusion coefficients for the electrolytes E_1_ and E_2_.

**Table 2:** Diffusion Coefficient Values of I_3_^–^ Calculated from the Data of Diffusion-Limited Current Density Obtained by Linear Sweep Voltammetry

electrolyte/symbol	*C*_I_3_^–^_ (mol L^–1^)	*J*_lim_ (0.05 V/s) (mA cm^–2^)	*D*_I_3_^–^_ (cm^2^ s^–1^)
E_1_	0.02	1.38	1.79 × 10^–5^
E_2_	0.04	2.22	1.44 × 10^–5^
E_3_	0.06	1.14	4.92 × 10^–6^
E_4_	0.08	0.55	1.78 × 10^–6^
E_5_	0.1	0.53	1.37 × 10^–6^

The appropriate choice of electrolyte and the optimization of its chemical composition are critical factors for developing high efficiency and durable DSSCs. The main reasons for this importance include the electrolyte acting as a medium for ion transport between the photoanode and the counter-electrode of the DSSC.^50^ A high diffusion coefficient ensures ions move quickly and efficiently through the electrolyte, minimizing transport resistance and optimizing current generation. On the other hand, slow diffusion leads to the accumulation of ions near the electrode, which can cause the recombination of electrons and charge carriers (ions) within the cell, reducing conversion efficiency. This emphasizes the importance of optimizing electrolyte composition to balance ion mobility and reduce recombination losses, thereby enhancing the overall performance of DSSCs.^51^ For these reasons, a high diffusion coefficient for the electrolyte is essential to guarantee the efficient transport of ions and, therefore, the cell’s stable functioning.^52^

### 3.3 DSSC Characterization

**Figure 4:**
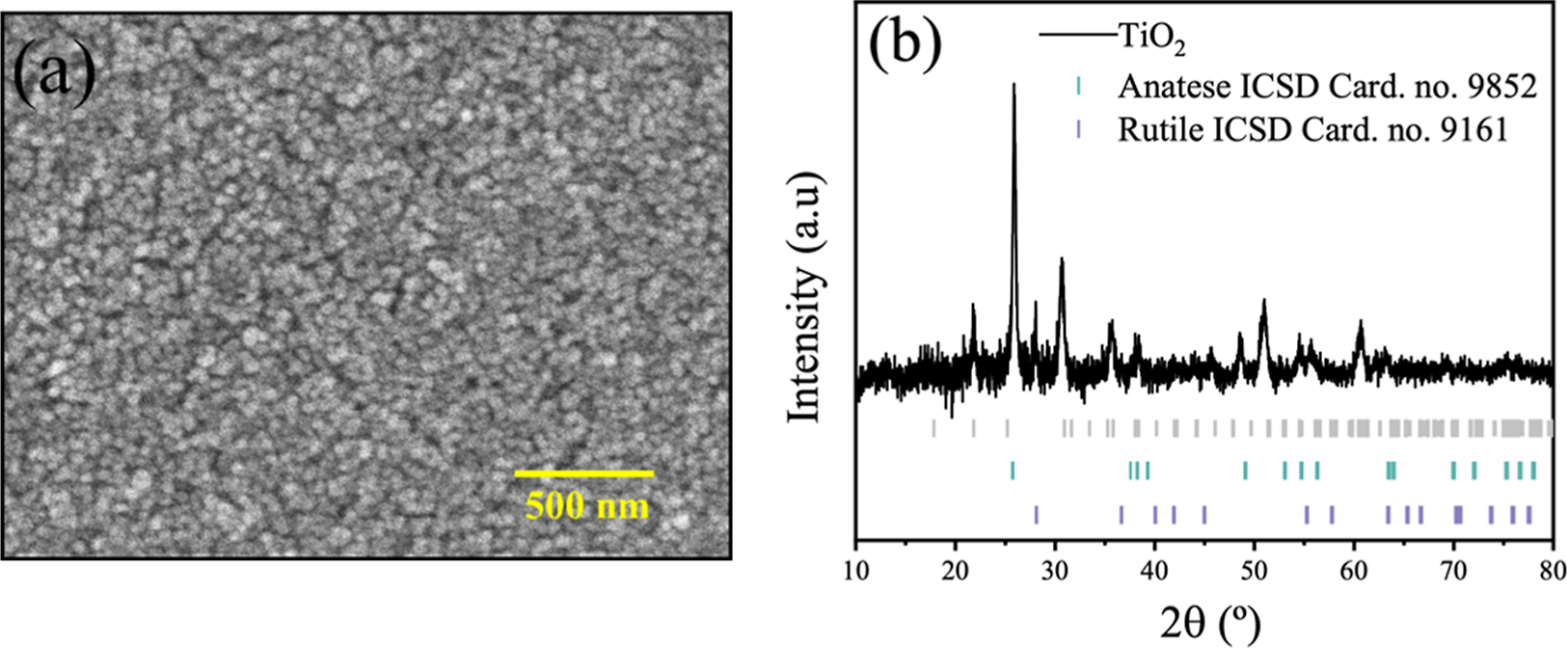
(a) SEM image of TiO_2_ film recorded and (b) XDR diffractogram of the TiO_2_ layer.

The morphology of TiO_2_ films was examined using scanning electron microscopy (SEM) at a magnification of 50,000×, Figure 4a. The commercial TiO_2_ paste applied as the semiconductor layer revealed a highly porous film structure with randomly oriented particles (Figure 4a). This porosity is critical for facilitating the effective diffusion of the dye and electrolyte into the film, which can positively impact cell performance.^53,54^ This roughness suggests more effective penetration and interaction of the Dye-CNDs complex within the porous matrix, potentially enhancing the overall performance of the sensitized film.

The structure of the TiO_2_ semiconductor layer was evaluated using X-ray diffraction (XRD) before the sensitization process and cell preparation. The analysis was conducted on a commercial TiO_2_ film subjected to thermal treatment at 400 °C for 20 min. The resulting diffractogram, presented in Figure 4b, revealed a mixed-phase structure comprising anatase and rutile phases, with the predominant anatase phase. The characteristic diffraction peaks for the anatase phase were identified at 25.8° (101), 38° (112), 48.5° (020), 54–55° (015) and (211), 62.7° (204), 70.3° (220), and 75.1° (215). Peaks corresponding to the rutile phase were observed at 27.5° (110), 35.5° (001), and 56.7° (220), consistent with ICSD (Inorganic Crystal Structure Database) entries no. 9852 and 9161.^55,56^

**Figure 5:**
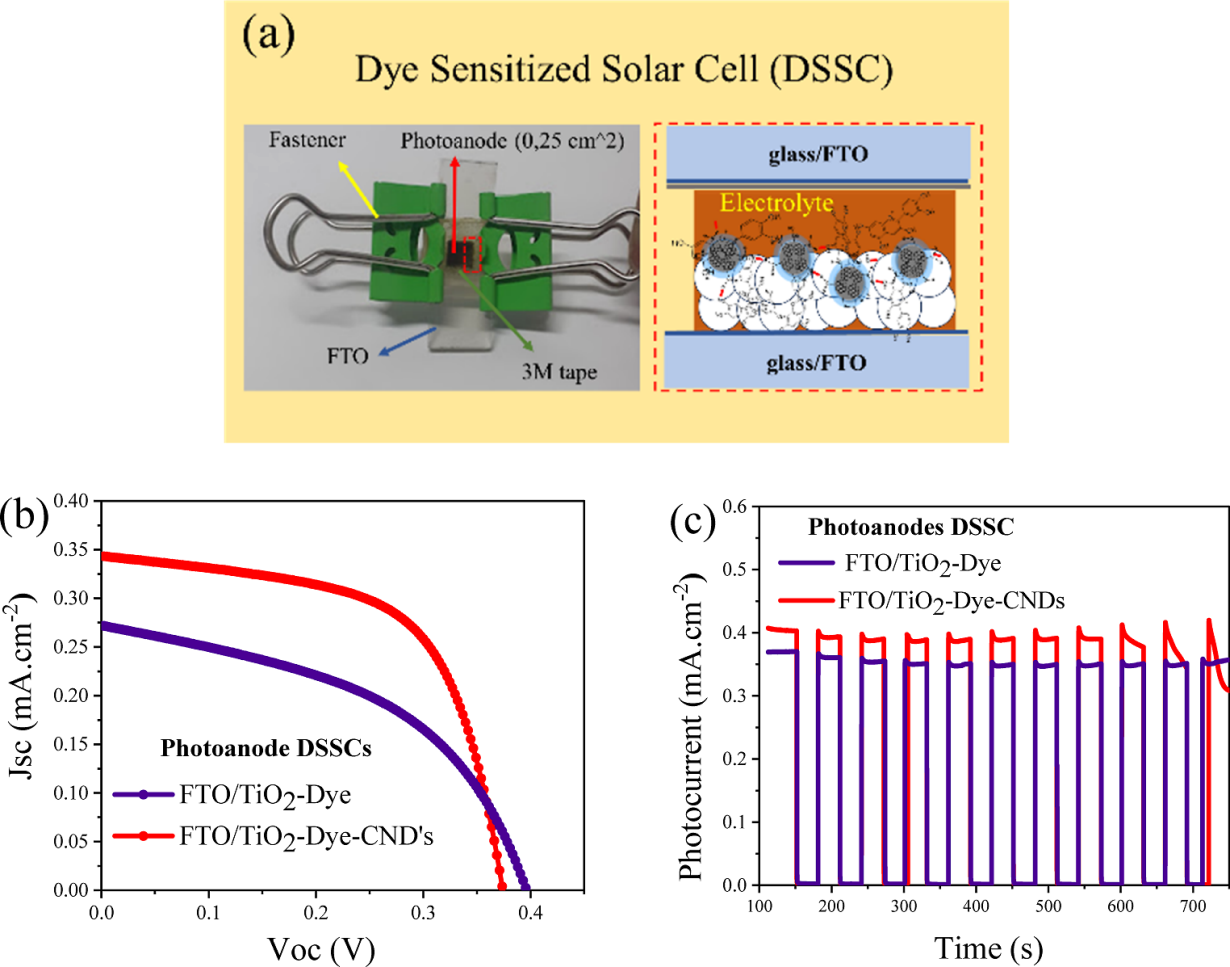
(a) Illustration of the DSSCs fabricated in this work; (b) current density–voltage curve; (c) of the DSSCs registered by chronoamperometry.

The cells were assembled using a standard dye-sensitized solar cell (DSSC) configuration in a layer-by-layer process, as illustrated in Figure 5a. Photoelectrochemical parameters were measured using a solar simulator system under standard light conditions of 100 mW cm^–2^ (AM 1.5G) and a potentiostat. The current density versus potential (*J*_sc_ vs *V*_oc_) curves and the photocurrent response over time are depicted in Figure 5b,c. The corresponding photoelectrochemical parameters for the cells are summarized in Table 3.

**Table 3:** Photoelectrochemical Parameters of DSSCs

photoanode DSSCs	area (cm^2^)	*J*_sc_ (mA/cm^2^)	*V*_oc_ (V)	FF (%)	efficiency η (%)
FTO/TiO_2_-Dye-CNDs	0.25	0.343 ± 0.13	0.373 ± 0.01	0.60 ± 0.02	0.10 ± 0.02
FTO/TiO2-Dye		0.272 ± 0.05	0.395 ± 0.01	0.46 ± 0.02	0.05 ± 0.01
FTO/TiO2-N719^61^	1.0	10.89	0.672	65	4.81

The power conversion efficiency (η) for the FTO/TiO_2_-Dye-CNDs photoanode cell was 5% higher than the simple photoanode (FTO/TiO_2_-Dye). The recorded open-circuit voltage (*V*_oc_) values were 0.373 V for the TiO_2_-Dye-CNDs photoanode cell and 0.395 V for the TiO_2_-Dye cell, showing a modest 2.2% difference between the cells. Conversely, the short-circuit current density (*J*_sc_) values were 0.343 mA/cm^2^ for the FTO/TiO_2_-Dye-CNDs photoanode and 0.272 mA/cm^2^ for the TiO_2_-Dye cell. The slight variance in *V*_oc_ can be attributed to the shift in the Fermi energy (*E*_F_) to lower values, which occurs due to the integration of CNDs within the TiO_2_ film. This shift in the Fermi level suggests an improved charge separation efficiency, potentially leading to enhanced photocurrent generation in the cell containing CNDs.^57^ This shift in the Fermi energy (*E*_F_) causes the energy difference between the redox pair and *E*_F_ to result in a lower *V*_oc_ value since *V*_oc_ is determined by the difference between *E*_F_ and the energy of the redox pair I^–^/I_3_^–^ (*E*_red_).^58,59^ As *E*_F_ moves closer to *E*_red_, the potential difference can be harnessed to decrease electrical work, resulting in lower *V*_oc_, highlighting how material modifications at the photoanode can impact the cell’s electronic properties and overall performance.

In contrast, the FTO/TiO_2_-Dye-CNDs photoanode cell exhibited a 7.1% increase in short-circuit current density (*J*_sc_) compared to the FTO/TiO_2_-Dye photoanode cell, with *J*_sc_ values of 0.343 ± 0.13 and 0.272 ± 0.05 mA·cm^–2^, respectively. This enhancement in *J*_sc_ can be attributed to the improved harnessing of incident sunlight,^45^ leading to more efficient charge photoinjection and enhanced charge transport due to CNDs. Additionally, CNDs can introduce trap states within the composite material,^60^ mitigating rapid electron recombination with photogenerated holes or the triiodide (I_3_^–^) species present in the electrolyte, thereby further contributing to overall cell performance improvements.

The photocurrent stability of the cells was evaluated by chronoamperometry measurements, applying *V* = 0 and *t* = 700 s in cycles of light and dark, alternating every 30 s. Photocurrent evaluation can be seen in Figure 5c. Generally, the photocurrent density of the cells remained stable throughout the measurement time. FTO/TiO_2_-Dye-CNDs photoanode cells showed a loss of photocurrent stability toward the end of the measurement. However, here, the photocurrent stability was a positive point and a strong indication that the adjusted concentration of I_2_ in the electrolyte substantially contributed to the stability of the DSSCs.

Sharma et al. (2023)^61^ employed the standard N719 dye as a sensitizer for TiO_2_, achieving comparable photoconversion efficiencies, Table 3. These results underscore the promise of natural dyes combined with CNDs. Moreover, although synthetic dyes provide advantageous properties and relatively higher efficiencies, their dependence on rare and heavy metals introduces environmental and health risks, underscoring the need to shift toward dyes with a lower environmental impact.

### 3.4 Energy Diagram for TiO_2_-Dye-CNDs and TiO_2_-Dye Photoanodes

To illustrate the photogenerated charge transfer processes following solar excitation, an energy diagram for the photoanode was constructed using complementary data from the current literature. As evidenced by Pfeifer and collaborators (2013),^62,63^ TiO_2_ has a conduction band (CB) edge and valence band (VB) edge of −4.0 and −7.2 eV, respectively. The highest occupied molecular orbital (HOMO) and lowest unoccupied molecular orbital (LUMO) levels of an anthocyanin molecule in water (specifically, a flavylium cation) are positioned at −5.88 and −2.33 eV, respectively.^64^

**Figure 6:**
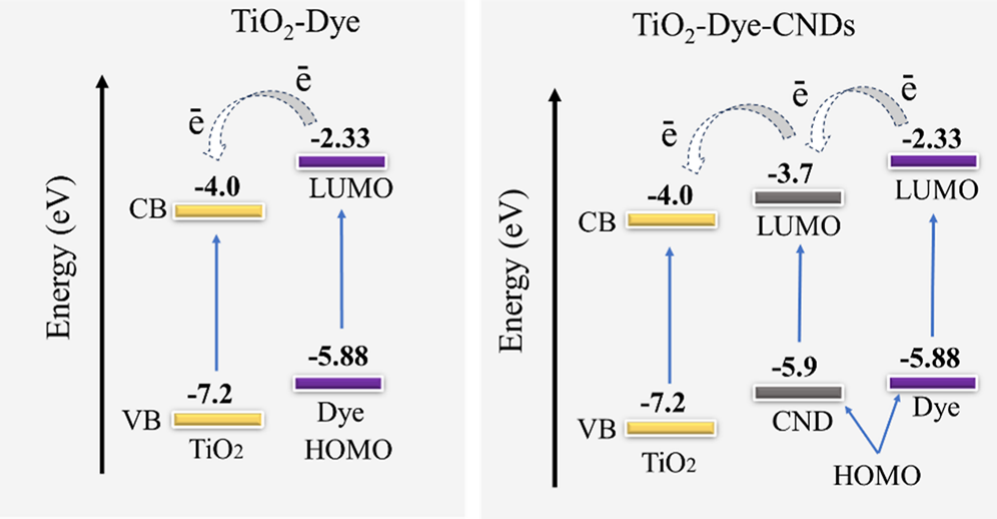
Energy diagram proposed is based on complementary information from current literature regarding the energy levels established for the VB and CB bands of TiO_2_,^63^ HOMO and LUMO of CNDs^65^ and natural dye.^66^

The focus on anthocyanin in this study stems from its abundant presence in the fruit of *L. australis*.^46^ Additionally, the HOMO and LUMO levels of carbon dots synthesized from similar raw materials are approximately −5.9 and −3.2 eV, respectively.^65^ Based on this information, an energy diagram for the photoanodes was proposed, indicating that the arrangement of the upper edges of the energy bands remains in thermodynamically favorable positions for efficient charge transfer processes. This diagram, depicted in Figure 6, illustrates the energy alignments for the TiO_2_-Dye-CNDs and TiO_2_-Dye photoanodes.

As illustrated in Figure 6, the LUMO levels of the anthocyanin molecule (−3.65 eV) and the CNDs (−3.2 eV) are positioned above the conduction band edge of TiO_2_ (−4.0 eV). This alignment creates a favorable driving force, enabling more efficient charge injection into the TiO_2_ conduction band. Nevertheless, charge recombination remains a challenge along this pathway, with the most likely recombination processes involving interaction with the photogenerated hole or triiodide (I_3_^–^) in the electrolyte, thereby impacting energy conversion efficiency.^23^ The observed *J*_sc_ values indicate that CNDs enhance the charge transfer process and mitigate recombination by introducing trap states.^67^ These trap states effectively act as barriers, preventing electrons from recombining prematurely and allowing them to reach the external circuit more efficiently.

In a typical DSSC, efficient electron movement requires the energy levels of the photoactive layer to align favorably with the TiO_2_ CB. Specifically, the LUMO of the CNDs, must be positioned above the CB edge of TiO_2_.^68,69^ Upon light absorption, photoexcited electrons from the dye or Dye-CNDs composite are injected into the TiO_2_ CB, initiating charge separation, and creating a potential difference. Once injected, electrons travel through the TiO_2_ crystal lattice toward the counter electrode, driven by the electric field. The electron’s departure from the dye or Dye-CNDs leaves behind a positive hole in the HOMO, which is subsequently neutralized by an electron from the redox pair in the electrolyte.^62^ This regeneration step is critical for sustaining the photoinduced charge generation cycle and enabling continuous electricity generation in the DSSC.

### 3.5 Electrochemical Impedance Spectroscopy for FTO/TiO_2_-Dye and FTO/TiO_2_-Dye-CNDs Photoanodes

**Figure 7:**
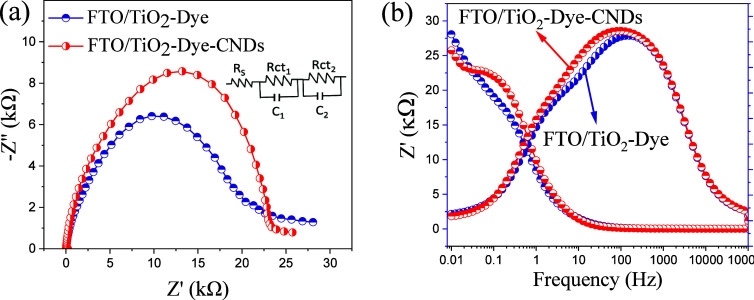
EIS of FTO/TiO_2_–Dye and FTO/TiO_2_-Dye-CNDs photoanodes under 100 mW cm^–2^ illumination: (a) Nyquist plot and (b) Bode plot.

EIS was applied to investigate the charge transfer process between the interfaces of the DSSC. Impedance profiles were recorded at open circuit voltage over a frequency range from 100 kHz to 0.01 Hz under standard illumination of 100 mW cm^–2^, modulating the overlaid sinusoidal potential wave (amplitude) frequency. The Nyquist and Bode plots, presented in Figure 7a,b, reveal a tendency for a double semicircle for both devices. However, this distinction of the arcs is partially masked due to the overlap of the larger semicircle (*R*_ct_2__) at medium frequency.

In Grätzel-type solar cells (DSSCs), three semicircles are typically expected in impedance spectroscopy analyses. The high-frequency semicircle corresponds to the charge transport resistance at the counter electrode/electrolyte interface (*R*_ct_1__), the medium-frequency semicircle represents the charge transport resistance at the photoanode/electrolyte interface (*R*_ct_2__), and the low-frequency semicircle is attributed to the electrolyte resistance (*R*_ct_3__).^70^ However, this study did not observe the third semicircle in the Nyquist and Bode plots, and this can be attributed to the low viscosity of the electrolyte, which facilitates ion diffusion and reduces the charge transfer resistance, rendering *R*_ct_3__ either imperceptible or overlapping with *R*_ct_2__.^71,72^

The semicircles were fitted with the equivalent circuit model (Figure 7a inset), and the resulting fitting parameters are in Table 4. R_S_ represents the series resistance (FTO sheet resistance), while *R*_ct_1__, *R*_ct_2__, and *R*_ct_3__ represent the charge transfer resistances at the CE/electrolyte, photoanode/electrolyte, and electrolyte interfaces.

**Table 4:** Parameters Obtained by Equivalent Circuit Model

parameters	FTO/TiO_2_-Dye	FTO/TiO_2_-Dye-CNDs
*R*_s_	25.07 Ω	24.27 Ω
*R*_ct_1__	12.7 kΩ	16.3 kΩ
*R*_ct_2__	16.9 kΩ	19.4 kΩ
*R*_ct_3__		

Simulation of the equivalent circuit provided insights into the charge transfer resistance (*R*_ct_2__) associated with the medium-frequency semicircle for both photoanodes, as detailed in Table 4. The results indicate that incorporating CNDs led to a modest increase in *R*_ct_2__, with a value of 19.4 kΩ compared to 16.9 kΩ for the CND free photoanode. This slight increase suggests that while the presence of CNDs introduces additional interfacial interactions, the overall charge transfer process remains efficient, albeit slightly less favorable than the CND free photoanode.

Equation 4 was used to evaluate the balance between charge collection and recombination processes in the cells. The ratio of recombination to transport parameters serves as a key metric for determining the charge collection efficiency in the TiO_2_ film, which is directly related to the electron diffusion length (*L*_n_).^24,73^*L*_n_ represents the average distance an electron travels through the film before recombining. In practical terms, if *L*_n_ is smaller than the thickness of the TiO_2_ film (*L*_n_ < *d*), only a limited fraction of the photogenerated charges injected into the TiO_2_ conduction band will be collected, reducing the overall efficiency. Conversely, achieving a diffusion length greater than the TiO_2_ film thickness (*L*_n_ > *d*) is desirable, as it ensures efficient charge collection and minimizes recombination losses.^24^ In the present work, the film thickness (*d*) of TiO_2_ was estimated at 60 μm, according to the manufacturer’s specifications for the Scotch tape Magic 810, used to delimit the area of the TiO_2_ film prepared by the doctor blade technique. The calculated *L*n values were 48.6 μm for the FTO/TiO_2_-Dye photoanode cell and 52.3 μm for the FTO/TiO_2_-Dye-CNDs photoanode cell. These results indicate that although efficient charge collection is not favored, since *L*_n_ < *d*, the presence of CNDs slightly favors capturing these photogenerated charges, which explains the higher *J*_sc_ presented.

## 4 Conclusion

This study successfully demonstrated the potential of photoluminescent carbon nanodots (CNDs) combined with natural dye extracted from *L. australis* (Pixirica) fruit as TiO_2_ sensitizers in Grätzel type solar cells (DSSCs). The synthesized CNDs exhibited characteristic absorption peaks at 260 and 340 nm, along with excitation wavelength dependent photoluminescence, as confirmed by UV–vis and photoluminescence analyses. The maximum emission was observed at 378 nm when excited at 380 nm.

The average size of the nanoparticles, as determined by TEM and DLS analyses, ranged between 5 and 30 nm. When incorporated, the composite sensitizer solution (Dye-CNDs) exhibited a red shift in the absorption spectrum, likely due to the formation of new energy states within the composite material, enhancing solar energy utilization. The study also investigated the influence of iodine concentration on the electrolyte composition, revealing that increased I_2_ concentrations significantly reduced the diffusion-limited current density, proportionally affecting the electrolyte’s diffusion coefficient.

Photoelectrochemical characterization of the cells indicated improved open-circuit voltage (*V*_oc_) and short-circuit current density (*J*_sc_) values for the FTO/TiO_2_-Dye-CNDs photoanode cell, suggesting that the presence of CNDs modifies the Fermi energy level and, consequently, alters the cell’s potential (*V*_oc_). Electrochemical impedance spectroscopy (EIS) measurements showed a moderate charge transfer resistance (*R*_ct_2__) increase for the FTO/TiO_2_-Dye-CNDs photoanode. Furthermore, the FTO/TiO_2_-Dye-CNDs photoanode exhibited a 7.1% increase in short-circuit current density (*J*_sc_), which can be attributed to the longer electron diffusion length (*L*_n_ = 52.3 μm). This enhancement contributed to a 5% improvement in the power conversion efficiency of the cell utilizing the Dye-CNDs composite compared to Dye only.

These findings underscore the promising role of carbon nanodots (CNDs) as enhancers of natural dye-based photosensitizers in Grätzel type solar cells. As an easily synthesized and cost-effective nanomaterial, CNDs hold significant potential for broader applications across various scientific fields. Future research should explore the impact of different oxidation states of CNDs to enhance DSSC performance further and optimize their photophysical properties.

## Data Availability

Data will be available upon request.
